# Left Ventricular Regional Wall Motion Abnormality is a Strong Predictor of Cardiotoxicity in Breast Cancer Patients Undergoing Chemotherapy

**DOI:** 10.5935/abc.20180220

**Published:** 2019-01

**Authors:** Márcio Vinícius Lins de Barros, Ariane Vieira Scarlatelli Macedo, Sebastian Imre Sarvari, Monica Hermont Faleiros, Patricia Tavares Felipe, Jose Luiz Padilha Silva, Thor Edvardsen

**Affiliations:** 1 Faculdade de Saúde e Ecologia Humana, Vespasiano, MG - Brazil; 2 Rede Materdei de Saúde, Belo Horizonte, MG - Brazil; 3 University of Oslo, Oslo - Noruega; 4 Universidade Federal do Paraná, Curitiba, PR - Brazil

**Keywords:** Ventricular Dysfunction, Left, Drug Therapy, Cardiotoxicity, Breast Neoplasms, Anthracyclines, Trastuzumab

## Abstract

**Background:**

Chemotherapeutic agents of anthracyclines class and humanized monoclonal antibodies are effective treatments for breast cancer, however, they present a potential risk of cardiotoxicity. Several predictors have been recognized as predictors in the development of cardiac toxicity, and the evaluation of left ventricular segmental wall motion abnormalities (LVSWMA) has not been studied.

**Objective:**

To analyze prospectively the role of LVSWMA among echocardiographic parameters in the prediction of development of cardiotoxicity in breast cancer patients undergoing treatment with chemotherapy.

**Methods:**

Prospective cohort of patients diagnosed with breast cancer and in chemotherapy treatment with potential cardiotoxicity medications including doxorubicin and trastuzumab. Transthoracic echocardiograms including speckle tracking strain echocardiography were performed at standard times before, during and after the treatment to assess the presence (or lack thereof) of cardiotoxicity. Cardiotoxicity was defined by a 10% decrease in the left ventricular ejection fraction, on at least one echocardiogram. Multivariate logistic regression models were used to verify the predictors related to the occurrence of cardiotoxicity over time.

**Results:**

Of the 112 patients selected (mean age 51,3 ± 12,9 years), 18 participants (16.1%) had cardiotoxicity. In the multivariate analysis using the logistic regression model, those with LVWMA (OR = 6.25 [CI 95%: 1.03; 37.95], p < 0,05), LV systolic dimension (1.34 [CI 95%: 1.01; 1.79], p < 0,05) and global longitudinal strain by speckle tracking (1.48 [CI 95%: 1.02; 2.12], p < 0,05) were strongly associated with cardiotoxicity.

**Conclusion:**

In the present study, we showed that LVWMA, in addition to global longitudinal strains, were strong predictors of cardiotoxicity and could be useful in the risk stratification of these patients.

## Introduction

The introduction of new chemotherapeutic agents, and the use of advanced and precise radiotherapy techniques in the last decades have dramatically improved breast cancer survival.^1^ Chemotherapeutic drugs of the anthracycline class, and the humanized monoclonal antibodies, such as trastuzumab, are widely used and highly effective agents for breast cancer treatment.^2^ Unfortunately, anthracyclines can induce cardiotoxic effects, and the severity of these adverse effects is compounded by concomitant use of trastuzumab.^3^

Chemotherapy may induce numerous cardiovascular complications, including hypertension, congestive heart failure, thromboembolic diseases, ischemic heart disease, QT prolongation, and bradycardia.^3^ When used in combination, anthracyclines and trastuzumab may result in heart failure in up to 27% of patients.^4^ Among cancer survivors, a third will die of cardiovascular disease. Thus, the need for optimal cardiac care in the cancer population has become evident. Early detection of cardiac dysfunction may allow implementation of cardioprotective strategies before potentially irreversible myocardial damage has occured.^5^

The definition of cancer therapy-related cardiac dysfunction (CTRCD) is based on a serial decline in left ventricular (LV) ejection fraction (EF). Two-dimensional echocardiography (2DE) is increasingly used for monitoring cardiac function during cancer treatment due to its widespread availability and safety. Echocardiography allows assessment of systolic and diastolic function, pulmonary pressures, valvular function, right ventricular function, and the pericardium.^6^

Reduction in LV EF likely occurs late in the natural history of CTRCD patients as reduction in LV EF may not be overt until a substantial amount of myocardial reserve has been exhausted, therefore more sensitive screening modalities for LV dysfunction are needed. Despite the recognition of several echocardiographic parameters associated with CTRCD, including novel echocardiography-derived parameters of myocardial mechanics, such as strain and strain rate, currently there is no consensus in the medical practice to fully predict which patients are prone to develop cardiotoxicity.^6^^-^^8^ Previous studies have demonstrated the presence of regional myocardial dysfunction in patients with CTRCD,^9^^-^^11^ however its role as a risk predictor has not been established. The purpose of this study is to verify the association between the occurrence of LV segmental wall motion abnormality and the development of cardiotoxicity in patients with breast cancer undergoing chemotherapy.

## Methods

### Study population

This study is part of a prospective cohort study of patients with breast cancer recruited from the Mater Dei Hospital in the city of Belo Horizonte - MG from January 2010 through December 2016. Inclusion criteria were, age above 18 years, histologically confirmed breast cancer diagnosis, treatment with doxorubicin and/or trastuzumab, and who underwent echocardiography, according to the rules of the hospital protocol. Exclusion criteria were patients with previous diagnosis of ventricular dysfunction including regional wall motion abnormality, significant valve disease, congenital heart disease, arrhythmias, chronic coronary artery disease and left bundle branch block by electrocardiography. Treatment regimens were at the discretion of the oncologist and consisted of the use of the following drugs alone or in combination: 1) doxorubicin and cyclophosphamide; 2) paclitaxel; 3) trastuzumab. The dosages of the medications were prescribed according to guidelines.^12^

Clinical (e.g., hypertension, dyslipidemia, diabetes) laboratorial (e.g., sodium, potassium, calcium, magnesium, hemoglobin, creatinine and BNP) and transthoracic echocardiograms were collected at baseline and standardized time intervals for each treatment regimen, 6 months after treatment completion and annually thereafter.

### Echocardiography

All patients were referred to a transthoracic echocardiogram, including longitudinal strain assessment with two-dimensional speckle-tracking echocardiography (2D STE). The echocardiographic studies and analyses were performed by an experienced cardiologist (M.V.L.B.). The following echocardiographic parameters were assessed: LV end-systolic and end-diastolic diameters and left atrial diameter. LV ejection fraction was assessed using Simpson's biplane method. Visual assessment of regional myocardial function was assessed on the basis of the observed wall thickening and endocardial motion of the myocardial segment, as described previously.^13^ Abnormal septal motion was characterized as a atypical movement of the interventricular septum during cardiac cycle with a two-dimensional echocardiography-guided M-mode approach. Diastolic function was assessed and classified using published criteria.^14^ LV diastolic dysfunction was stratified into four grades as normal, impaired relaxation, pseudo normal filling or restrictive.

Longitudinal strain by 2D STE was obtained from apical four-chamber, two- chamber, and long-axis views. Three cardiac cycles from each view were recorded for offline analyses with a frame rate > 50 frames/sec. Peak negative longitudinal strain was assessed in 16 LV segments, defined as the peak negative value during the entire cardiac cycle, hence including post systolic shortening, and was averaged to global longitudinal strain (GLS). CTRCD was defined as a decrease in LVEF of > 10 percentage points, to a value < 53% at repeated cardiac imaging studies during follow-up after chemotherapy.^15^

The echocardiographic studies were performed at standardized intervals according to the treatment regimen. 1) Patients treated with anthracyclines without trastuzumab underwent an echocardiographic study at baseline, at completion of chemotherapy, and every six months after completed treatment. 2) Patients treated with anthracyclines and trastuzumab underwent an echocardiographic study at baseline, after completion of the anthracycline treatment regimen, every 3 months during trastuzumab therapy, and every six months after completed treatment. 3) Patients treated with trastuzumab without anthracyclines underwent an echocardiographic study at baseline, every 3 months during trastuzumab therapy, and every six months after completed treatment.

Echocardiographic assessment was completed in patients with at least three echocardiographic studies performed during the research period.

### Statistical Analysis

To describe the qualitative variables, the absolute and relative frequencies were used, while to describe the quantitative variables, measures of central tendency, dispersion and position were used.

In order to identify the factors that influenced the occurrence of cardiotoxicity over time, the Generalized Estimation Equations (GEE) approach was used. An exchangeable correlation structure was assumed for the repeated observations of the same individual. Univariable and multivariable models with a logit link function were considered. There was no occurrence of cardiotoxicity at the first measurement occasion and therefore we also included the baseline values of the time-dependent predictors. Missing values were excluded from the analyses. Variables that were statistically significant at the 0.20 level were included in the multivariable model. For this final model, a level of significance of 0.05 was adopted. Reproducibility of visual assessment of abnormal regional myocardial function was evaluated by the kappa statistics.

ROC curves were built and the discrimination ability of the model was assessed by the area under the ROC curve. All statistical analysis was performed using R Statistical Software 3.4.1 and the R packages gee, pROC and PredictABEL.

### Ethical considerations

The study complies with the Declaration of Helsinki and was approved by the Research and Ethical Council of the Mater Dei Hospital.

## Results

### Studied population

A total of 112 patients were included. Mean follow-up time was 491 days. The characteristics of the population studied are summarized in Table 1. Most of the patients in the cohort were female (98.2%). Mean age was 51.3 ± 12.9 years.

**Table 1:** Clinical and laboratorial characteristics of 112 patients undergoing chemotherapy

Variable	n
Age (mean ± SD)	51,4 ± 11,1
Female (n/%)	111 (99,1%)
BMI (kg/m^2^)	26,1 ± 5,8
Mastectomy (n/%)	111 (99,1%)
Median follow-up time (months)	16
Radiotherapy (n/%)	74 (66)
**Chemotherapy (n/%)**	
AC-T	90 (80)
Anti HER2	29 (26)
Others	20 (18)
Hormone Therapy (n/%)	72 (64)
**Cardiovascular risk factors (n/%)**	
Hypertension	39 (35)
Diabetes	8 (7)
Hyperlipidemia	21 (19)
Smoking	25 (22)

BMI: body mass index; AC-T: Doxorubicin/cyclophosphamide - Taxol (Paclitaxel).

Of the 112 patients followed up, 18 (16.1%) presented CTRCD.

**Figure 1:**
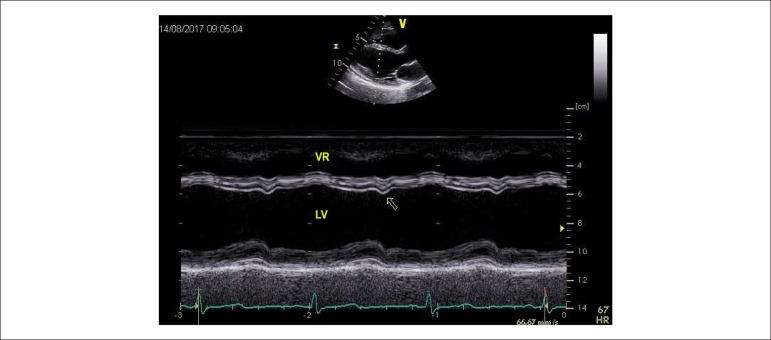
Two-dimensional echocardiography-guided M-mode showing abnormal motion of interventricular septum (arrow) during chemotherapy treatment. LV: left ventricle; RV: right ventricle.

The characteristics of the patients with abnormal LV segmental wall motion are summarized in table 2. LV segmental wall motion abnormality was found in 16 (14%) patients, most commonly at the time of the second echocardiographic study (43%). LV segmental wall motion analyses by visual assessment showed abnormalities most frequently in the interventricular septum (78.5% - Figure 1), the inferior (14.3%), and the inferolateral (7.1%) walls. During the follow-up, no patient presented left bundle branch block by electrocardiography study.

**Table 2:** Characteristics in patients with segmental wall motion abnormality during chemotherapy

Patient	Age	Treatment*	Abnormal contraction	Echocardiographic follow-up	Risk factors	CTRCD	Follow-up
2	49	1,2,	Infero-septal Hypokinesis	5	no	yes	Death
5	40	1,2	Abnormal Septal motion	2	no	yes	NYHA I
12	68	1,2	Ínfero-lateral Hypokinesis	5	no	yes	NYHA I
21	30	1,2	Abnormal Septal motion	3	dyslipidemia	no	
27	43	1,2	Abnormal Septal motion	2	no	no	
52	73	1,	inferior Hypokinesis	4	Diabetes, Hypertension	yes	NYHA I
63	53	1	Septal Hypokinesis	2	no	no	
67	77	1,2	Abnormal Septal motion	4	hypertension	yes	NYHA II
72	44	1,2	Abnormal Septal motion	2	no	no	
84	59	1,2	Inferior Hypokinesis	4	no	no	
88	34	1	Abnormal Septal motion	3	no	no	
92	39	1	Abnormal Septal motion	3	no	yes	death
100	41	1	Infero-septal Hypokinesis	2	no	no	
110	62	1	Septal hypokinesis	2	no	yes	NYHA !

* 1: anthracycline; 2: transtuzumab; CTRCD: cancer therapy-related cardiac dysfunction.

**Figure 2:**
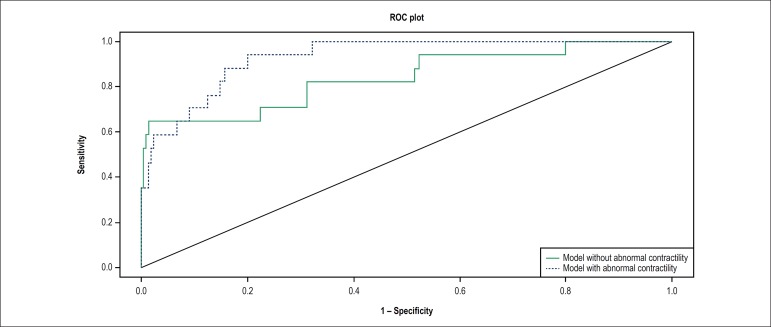
Roc curve of the multivariate model with and without evaluation of segmental abnormal contractility.

Among the variables studied, it was observed at multivariable analysis that GLS measurements as well as LV systolic dimensions and the presence of LV regional wall motion abnormalities at the baseline study could predict development of cardiotoxicity (Tables 3 and 4). The analysis of ROC curve of the final model (Figure 2) showed an area under the curve (AUC) of 0.93 (0.88 - 0.98). When we exclude the presence of wall motion abnormality in the model, the AUC was 0.84 (0.72-0.96) showing additive predictive power of this variable (p = 0.047). Intraobserver variability and interobserver variability for wall motion assessment were 0.89 and 0.81, respectively.

**Table 3:** Univariate analyses of predictors related of cardiotoxicity

Variable	O.R.	95% CI	p
Age	1.03	[0.99; 1,07]	0.151
LVDD	1.22	[0.99; 1.50]	0.061
LVSD	1.69	[1.35; 2.12]	0.000
Diastolic dysfunction	3.55	[1.34; 9.44]	0.011
Regional wall motion abnormality	8,91	[2.75; 28.82]	0.000
LA	0.95	[0.78; 1.16]	0.624
GLS	1.96	[1.25; 3.09]	0.022
PASP	1.86	[0.32; 10.99]	0.491
BNP	1.24	[00.91; 1.70]	0.306
Troponin	3.36	[0.49; 23.14]	0.219
Creatinine	0.04	[0.00; 2.20]	0.113
Hemoglobin	0.90	[0.60; 1.35]	0.608
Sodium	1.01	[0.98; 1.03]	0.623
Potassium	1.33	[0.51; 3.51]	0.559
Calcium	1.06	[0.81; 1.39]	0.657
Magnesium	6.20	[0.67; 57.73]	0.204
Hypertension	0.79	[0.26; 2.39]	0.673
Dyslipidemia	0.41	[0.07; 2.21]	0.298
Diabetes	1.15	[0.15; 8.83]	0.894

LVDD: left ventricular diastolic dimension; LVSD: left ventricular systolic dimension; GLS: global longitudinal strain; LA: left atrium dimension; PASP: pulmonary artery systolic pressure; BNP: brain natriuretic peptidium.

**Table 4:** Multivariate analysis of predictors related of cardiotoxicity

Variable	O.R.	95% CI	p
LVSD	1.34	[1.01; 1.79]	0.044
Regional wall motion abnormality	6.25	[1.03; 37.95]	0.046
GLS	1,48	[1,02; 2.12]	0.036

LVSD: left ventricular systolic dimension; GLS: global longitudinal strain.

## Discussion

In this prospective, longitudinal cohort study, we showed that the presence of regional wall motion disturbance and decreased GLS are strong predictors of CTRCD.

Earlier histopathological studies performed from endomyocardial biopsies have demonstrated an initially focal and dispersed involvement of myocytes, surrounded by normal cells in patients treated with anthracyclines.^16^ As the toxicity evolves, the frequency of these alterations increases, leading to significant myocardial damage and later on to diffuse myocardial fibrosis. Thus, segmental contractile dysfunction may precede the intense and diffuse involvement of the heart seen in CTRCD. In this context, interventricular septum dyssynchrony, as well as segmental hypokinesia may be present due to tissue edema and/or focal cellular damage.^17^

Indeed, Piotrowsk et al.^9^ demonstrated that in 60.9% of patients with LV systolic dysfunction regional wall motion abnormalities were observed in the first echocardiography that revealed a significant drop of LVEF. In the majority of these cases (64%), regional hypokinesis involved the interventricular septum.^9^ Previous studies using tissue Doppler and 2D strain have also shown regional contractile alterations in patients treated with chemotherapy.^10^^,^^11^ Boyd et al.^18^ demonstrated that in the group with subclinical LV dysfunction (> 11% reduction in GLS compared to before therapy) 58% of regional segments had a reduction in strain by > 11%, compared to 29% of regional segments in the group without subclinical LV dysfunction (p < 0.001).^18^

It is well known that reduction of longitudinal strain is an early predictive factor of cardiotoxicity induced by treatment with anthracyclines and trastuzumab, as confirmed by our results. Negishi et al. showed that GLS was an independent predictor of subsequent reductions in EF, with a discrimination improvement by adding GLS of -18.6% to traditional parameters by echocardiography in patients at risk for trastuzumab-induced cardiotoxicity.^19^ In another study, Sawaya el al.^20^ showed that in patients with breast cancer treated with chemotherapy, GLS measured at the completion of anthracycline therapy was useful in the prediction of subsequent cardiotoxicity.^20^

It was shown in a systematic review that an early reduction of 10% to 15% in GLS was a useful parameter for the prediction of cardiotoxicity.^21^ A small cohort study was associated with subclinical LV dysfunction as early as 1 week after treatment, showing a significant decrease in GLS and annular systolic velocity of the lateral LV wall 7 days after by trastuzumab treatment.^22^ Fei et al.^23^ found, in a cohort of 95 patients treated with anthracycline and trastuzumab, and followed for a mean time of 17 months, 20% with cardiotoxicity, demonstrating a significant association between GLS reduction and LVEF decline.^23^

The presence of diastolic dysfunction was not an independent predictor of CTRCD in our study. The use of diastolic dysfunction as a surrogate marker for predicting trastuzmab-induced cardiotoxicity is controversial. Earlier studies have shown that diastolic impairment of the LV occurs before deterioration in LV EF in anthracycline^24^^,^^25^ and transtuzumab^26^^,^^27^ induced cardiotoxicity. Development of diastolic dysfunction has been reported in up to 57% of patients after treatment with anthracyclines or anthracyclines plus trastuzumab.^28^ Cochet et al.^28^ Serrano et al.^29^ evaluated MUGA-derived diastolic parameters and found that impaired LV diastolic function before treatment was an independent predictor of trastuzumab-mediated cardiotoxicity. Boyd at al.^18^ showed in a cohort involving 140 patients followed for seven days that LV diastolic dysfunction grade significantly increased from 46% to 57% (p < 0.001) after treatment with anthracyclines. Importantly, diastolic dysfunction was more prevalent in the subgroup with a significant reduction in GLS, demonstrating the close association between systolic and diastolic dysfunction.^18^ A study using MUGA-derived diastolic function parameters investigated whether impairment of systolic function was preceded by diastolic dysfunction in a group of 77 female breast cancer patients undergoing trastuzumab therapy. The results of this study showed a nearly even number of patients with diastolic dysfunction preceding systolic dysfunction (54%), as compared to the number of patients with the opposite order (42%).^30^ Discrepancy among those studies is probably related to the different designs and interpretation of the results.

### Limitations

All patients were recruited from one center and the study consisted of a limited number of patients. The study was limited by a short duration of patient follow-up, and therefore any possible long term impact of the early echocardiography abnormalities are uncertain. Long term follow up is therefore necessary to determine the significance of these early observations. The proposed treatment was individually defined, including the use of cardio-protective drugs, which may have influenced our results.

## Conclusion

In this prospective cohort of 112 patients undergoing treatment with chemotherapy for breast cancer, we found segmental wall motion abnormality to be a strong predictor of cardiotoxicity. Therefore, assessment of segmental wall motion might be a useful tool in the evaluation of patients at risk of developing CTRCT, resulting in early detection of myocardial dysfunction and potential reduction in morbidity and mortality in these patients.
